# Validation of the Arabic version of the Muscle Dysmorphic Disorder Inventory (Ar-MDDI) among Lebanese male university students

**DOI:** 10.1186/s40337-023-00737-8

**Published:** 2023-01-26

**Authors:** Feten Fekih-Romdhane, Georges Merhy, Verginia Moubarak, Jinbo He, Radoslaw Rogoza, Rabih Hallit, Sahar Obeid, Souheil Hallit

**Affiliations:** 1 Department of Psychiatry “Ibn Omrane”, The Tunisian Center of Early Intervention in Psychosis, Razi Hospital, 2010 Manouba, Tunisia; 2 Faculty of Medicine of Tunis, Tunis El Manar University, Tunis, Tunisia; 3 School of Medicine and Medical Sciences, Holy Spirit University of Kaslik, P.O. Box 446, Jounieh, Lebanon; 4 School of Humanities and Social Science, The Chinese University of Hong Kong, Shenzhen, 518172 Guangdong China; 5 Cardinal Stefan Wyszyński University in Warsaw, Warsaw, Poland; 6 Social Innovation Chair, University of Lleida, Lleida, Spain; 7 Social and Education Sciences Department, School of Arts and Sciences, Lebanese American University, Jbeil, Lebanon; 8 Psychology Department, College of Humanities , Effat University, Jeddah, 21478 Saudi Arabia; 9 Applied Science Research Center, Applied Science Private University, Amman, Jordan; 10 Research Department, Psychiatric Hospital of the Cross, Jal Eddib, Lebanon

**Keywords:** Muscle dysmorphia, Arabic, Validation, Confirmatory factor analysis

## Abstract

**Background:**

To date, the vast majority of research on disordered eating symptomatology and body image disturbances from the Arab world have been performed exclusively among women; and mainly used thinness-oriented measures that are not sensitive to detect muscularity-oriented symptoms, which are more evident in males. Therefore, the objective of our study was to validate the Arabic version of the Muscle Dysmorphic Disorder Inventory (Ar-MDDI), in order to make it accessible for Arabic-speaking populations.

**Methods:**

Using a snowball sampling technique, men university students (n = 396) from multiple universities in Lebanon filled the survey in this cross-sectional designed study (January–May 2022). A soft copy of the questionnaire was created using google forms software, and sent to participants through the different social media platforms such as Facebook, Instagram, and WhatsApp. We used the Muscle Dysmorphic Disorder Inventory to assess Muscle Dysmorphia, along with the Big Three Perfectionism Scale to assess perfectionism and Eating Attitude Test (EAT) to evaluate the inappropriate eating attitudes. To explore the factor structure of Ar-MDDI, we computed a principal-axis Exploratory Factor Analysis (EFA) with the first split-half subsample using the FACTOR software. We used data from the second split-half to conduct a Confirmatory Factor Analysis (CFA) using the SPSS AMOS v.29 software. Pearson correlation test was used to test the convergent and divergent validity of the Ar-MDDI scale with the other scores included in the study.

**Results:**

The results of the EFA revealed three factors, which explained 57.68% of the common variance: Factor 1 = Appearance intolerance, Factor 2 = Drive for size, and Factor 3 = Functional impairment. The CFA fit indices of the three-factor model of the Ar-MDDI scale showed good results. Moreover, 254 (64.1%) of the participants had inappropriate eating attitudes (EAT scores ≥ 20). Indices suggested that configural, metric, and scalar invariance was supported according to eating attitudes. No significant difference between participants with appropriate versus inappropriate eating attitudes in terms of functional impairment, drive for size and appearance intolerance. Perfectionism scores correlated positively with the Ar-MDDI, which suggests divergent validity.

**Conclusion:**

Our findings revealed that the validation of the Arabic scale yielded excellent properties, preliminarily supporting its use for the assessment of muscle dysmorphia among Arabic-speaking university men. This would hopefully allow for its timely detection and management in Arab clinical settings and encourage cross-cultural research on this topic.

**Supplementary Information:**

The online version contains supplementary material available at 10.1186/s40337-023-00737-8.

## Plain English Summary

Muscle dysmorphic disorder (MDD) refers to an important muscularity-oriented dimension related to eating disorder (ED) symptoms and body image disturbances. One widely used self-report measure to assess the MDD construct is the Muscle Dysmorphic Disorder Inventory (MDDI). Despite its high clinical relevance, MDD has not been previously investigated in the Lebanese context and among the Arabic-speaking communities in general. Indeed, the multiple scales assessing ED symptomatology that have previously been translated to the Arabic language and adapted to the Lebanese population have been mainly focused on thinness-oriented ED; and no Arabic validation of muscularity-oriented body image and disordered eating symptoms measures, including the MDDI, exists so far to the best of our knowledge. To overcome this gap, the present study aimed to validate the Arabic version of the MDDI (Ar-MDDI) in a sample of Lebanese men university students. The findings showed that the AR-MDDI is robust in terms of reliability, factor structure, convergent and divergent validity. We, therefore, preliminarily recommend its use in clinical practice, preventive interventions and future research in Arab settings.

**Supplementary Information:**

The online version contains supplementary material available at 10.1186/s40337-023-00737-8.

## Background

Traditionally, research on eating disorder (ED) symptoms, weight, and body image has mainly focused on women populations [1]; while men have for a long time remained largely under-researched [2, 3]. However, in recent years growing evidence has accumulated on occurrence of ED symptoms in men [2–7], which attracted increasing attention from researchers around the world [8]. Disordered eating behaviors and body image issues have different characteristics and features across genders [9], manifesting as a drive for muscularity and leanness (body composition) in men versus a drive for thinness in women [9, 10]. Indeed, men became increasingly affected by sociocultural body ideals, that may in turn result in the development of body dissatisfaction [11, 12] and muscularity-oriented symptoms [4]. It is of note, however, that the vast majority of previous literature on ED pathology and body dissatisfaction have been performed in Western societies due to an overrepresentation of the problem in these societies [2]. Although there has been a recent rise in ED symptoms prevalence in the Arab world [13, 14], only a dearth of research has been conducted in this region. Recent meta-analyses have even shown inverted patterns of prevalence rates due to a westernization expansion, with increased risk of ED among participants from non-Western cultures as compared to those from Western cultures [15, 16]. Particularly in Lebanon, studies documented high prevalence rates of ED symptoms in clinical populations (estimated as 46.1% for bulimia nervosa, 39.4% for anorexia nervosa, and 14.4% for binge eating) [17]. Lebanese studies in non-clinical populations also showed alarmingly high prevalence of ED pathology, such as orthorexia nervosa tendencies and behaviors (75.2%) [18], restrained eating (48.3%) [19] and disordered eating attitudes (23.8–25.3%) [20] in healthy adults, and body dissatisfaction in adolescents (45.1%) [21]. Another Lebanese study found that 36% of university students were slightly to extremely worried about their body image [22]. Hoteit et al. found that 22.5% of health science students and healthcare practitioners in Lebanon were at high risk of ED [23]. Significant gender differences have also been noted in the prevalence and correlates of a range of ED symptoms in the Lebanese general population (e.g., [24–26]). Despite this evidence, the existing literature on ED in the Arab world in general, and Lebanon in particular, is hindered by two major limitations. The first limitation lies to the fact that, to date, the vast majority of research on disordered eating symptomatology and body image disturbances have been performed exclusively among women [27]. The second limitation is that, although several scales assessing ED symptomatology have been translated to the Arabic language and adapted to the Lebanese population, these scales have been mainly focused on thinness-oriented ED, such as the Dutch Restrained Eating Scale [28], the ORTO-R [29, 30], the Dusseldorf Orthorexia Scale [31], the Teruel Orthorexia Scale [32], and the Eating Attitude Test (EAT-7 [33], EAT-26 [20]). These measures are not sensitive to detect ED symptoms in men, of a muscularity-oriented nature [9].

The present paper focuses on an important muscularity-oriented dimension related to ED symptoms and body image disturbances, muscle dysmorphic disorder (MDD). MDD has been listed in the Diagnostic and Statistical Manual-5 (DMS-5) as a variant of body dysmorphic disorder [34]. The disorder refers to an obsessive preoccupation with muscles’ size and shape [35], perceived as small, unattractive, and/or not sufficiently muscular and lean [36–39]; which often leads to a desire to hide one’s body [35] and avoid social situations [35, 40, 41], as well as a great shame [38], and substantial social and occupational impairments [35].

Overall, MDD has been shown to have numerous health consequences (for review, see [42]). More specifically, it was found to be significantly associated with high perfectionism [42]. The negative consequences highlight the crucial necessity for adequate treatment. It is clear that a timely recognition and diagnosis of MDD plays a major role in its effective management [43]; however, some evidence has shown that men-specific eating and body dysmorphic disorders remain largely unrecognized, undetected and untreated [36, 44, 45]. This is exacerbated by men's reluctance to seek help, partially because of stigma [46]. Therefore, early screening and detection are key to intervening before symptoms become disabling and therefore reducing delays to care attributable to stigma in this specific population.

Several self-report measures have been developed to evaluate muscularity-oriented body image and disordered eating symptoms. Among them, the most commonly used is the Muscle Dysmorphic Disorder Inventory (MDDI). The MDDI [47] is an instrument consisting of 13 items on a 5-point Likert type scale (from 1 “never” to 5 “always”), and has three subscales: appearance intolerance, drive for size, and functional impairment. The latter subscale is considered a key diagnostic indicator of MDD that other measures (such as the MASS (Muscle Appearance Satisfaction Scale) or the MDI (Muscle Dysmorphic Inventory)) omit to assess [42]. MDDI has been translated in different languages and countries, including Spanish [48], Portuguese [49], Turkish [50], and Chinese [51]. However, no Arabic validation has been done so far to the best of our knowledge. This has led to a lack of research on this topic in Lebanese and the broader Arabic-speaking communities; which strongly supports the need for making instruments assessing men-specific ED symptoms available for Arabic-speaking men in Lebanon and other parts of the world. A recent literature review by Melisse et al. concluded that the existing research on ED symptoms in Arab countries “suffers from potential limitations due to the use of non-validated assessment tools”, and called for more validation studies [52].

To address the above-mention limitations, this study aimed to translate and validate the MDDI into the Arabic language, by examining its internal consistency, factorial structure, convergent and divergent validity in a sample of Lebanese men university students. We expected that (1) the Arabic version scale would have good internal consistency; (2) the MDDI would have a three-factor structure; and (3) the total scores of the scale would be significantly correlated with disordered eating attitudes and perfectionism scores, indicating both its convergent and divergent validity. Evidence for the convergent validity of the MDDI through significant strong positive correlations with ED measures has previously been demonstrated (e.g., [53]). Additionally, perfectionism has been shown as a consistent risk factor for developing muscle dysmorphia [54]; and has, therefore, been chosen as a relevant construct for divergent validity.

## Methods

### Participants

University men (N = 396) from multiple universities in Lebanon were invited to fill the survey. Any student enrolled in any educational level (bachelor, master or PhD) was invited to fill the survey. Participants had a mean age of 25.63 years (SD = 5.84), ranging from 18 to 60 years and had a mean self-reported body mass index (BMI) 24.46 kg/m^2^ (*SD* = 3.51), ranging from 15.82 to 47.75 kg/m^2^. Most participants were single (79.0%).

Our sample was chosen using the snowball technique; a digital questionnaire was created using google forms software, and an online approach was conceived to proceed with the data collection (by sending the questionnaire through the different social media platforms such as Facebook, Instagram and WhatsApp). The study’s main aims and goals, in addition to instructions for filling the questionnaire, were conveyed online for the participants, prior to their participation. Later, initial participants were asked to recruit other participants they know, preferably as diverse as possible regarding place of habitat within the Lebanese governorates and within the same age interval required to participate in the study. There were no credits received for participation.

### Study design

One certified interpreter translated the MDDI scale from English into Arabic, while a second interpreter translated the Arabic version back into English. Next, the English version was assessed by a committee consisting of the research team and the two translators. There were very minor discrepancies (e.g., word choice differences) in the translation of the scale, which were resolved through consensus. The items of the MDDI scale in Arabic are presented in Additional file 1: Appendix 1.

Data collection took place between January and May 2022. Data was collected through the snowball technique, where the research team initiated the first contact with some participants by sending them the Google form link to the survey; those participants were solicited to send the link to other men they know. The link contained a brief introduction related to information about the study (e.g., objectives of the study, confidentiality of answers, estimated duration, etc.). Those who agreed to participate answered “yes” to the question related to their full willingness to participate in the study. The questionnaire took between 15 and 20 min to complete. Participation was voluntary, with no remuneration was given to any participant in return.

### Translation procedure

All scales, except the Eating Attitude Test, were first translated from English into Arabic by one psychologist, then back to English from Arabic by another psychologist. The Arabic version was verified by a linguistic professional. The principal investigator compared the English version to discern any discrepancies; all procedures were done according to the international recommendations of forward-back translation [55]. The questionnaire was pilot-tested on 20 participants before data collection. As all 20 participants could interpret the items correctly without difficulties, no changes were made. Thus, the translated scales were used in the present study.

### Measures

#### Sociodemographic information

We collected information about age, marital status and the self-reported height and weight to calculate the BMI.

#### Muscle dysmorphic disorder inventory

This scale is composed of 13 items, scored on a five-point Likert-type scale (0 = never to 4 = always) [47]. Three factors were previously identified: desire for size (DFS), appearance intolerance (AI), and functional impairment (FI). The Arabic items of the scale are available in Additional file 1: Appendix 1.

#### Big three perfectionism scale

This scale is composed of 16 items, scored on a five-point Likert scale (1 = strongly disagree to 5 = strongly agree) [56]. It yields three subscale scores: rigid perfectionism, self-critical perfectionism and narcissistic perfectionism. Higher scores reflect higher perfectionism in the three aspects. In this study, the Cronbach’s alpha values for the three scores were as follows: rigid perfectionism (McDonald’s ω = 0.91), self-critical perfectionism (McDonald’s ω = 0.88) and narcissistic perfectionism (McDonald’s ω = 0.84).

#### Eating attitude test

Validated in Lebanon [20], it is composed of 26 questions rated on a six-point Likert scale; higher scores reflect more inappropriate eating attitudes [57], specifically when participants score 20 and above on the test (McDonald’s ω = 0.97).

### Analytic strategy

#### Data treatment

There were no missing responses in the dataset. To examine the factor structure of the MDDI, we used an EFA-to-CFA strategy [58]. To ensure adequate sample sizes for both EFA and CFA, we split the main sample using the SPSS computer-generated random technique; the description of the two samples are shown in Table 1. There were no significant differences between the two subsamples in terms of mean age, *t*(394) = 0.02, *p* = 0.984, and BMI, *t*(393) = 1.81, *p* = 0.072.

**Table 1:** Items of the Ar-MDDI in English and Factor Loadings Derived from the Exploratory Factor Analyses (EFA) in the First Split-Half Subsample, and Standardised Estimates of Factor Loadings from the Confirmatory Factor Analysis (CFA) in the Second Split-Half Subsample

	EFA	CFA
Item		
Factor 1 = Appearance intolerance		
2	0.67	0.68
3	0.71	0.71
7	0.81	0.61
8	0.56	0.51
9	0.80	0.72
Factor 2 = Drive for size		
1	0.77	0.50
4	0.78	0.54
5	0.50	0.66
6	0.78	0.66
Factor 3 = Functional impairment		
10	0.78	0.69
11	0.77	0.70
12	0.79	0.85
13	0.74	0.74

#### Exploratory factor analysis (EFA)

To explore the factor structure of Ar-MDDI, we conducted a principal-axis EFA with the first split-half subsample using the FACTOR software [59]. We verified all requirements related to item-communality [60], average item correlations, and item-total correlations [61]. The Kaiser–Meyer–Olkin (KMO) measure of sampling adequacy (which should ideally be ≥ 0.80) and Bartlett’s test of sphericity (which should be significant) ensured the adequacy of our sample [62]. The procedure followed for determining the number of dimensions was the Parallel Analysis (PA) [63]. Weighted Root Mean Square Residual (WRMR) were also calculated to assess the model fit (values < 1 have been recommended to represent good fit) [64]. Item retention was based on the recommendation that items with “fair” loadings and above (i.e., ≥ 0.33) and with low inter-item correlations (suggestive of low item redundancy) as indicated by the anti-image correlation matrix should be retained [65].

#### Confirmatory factor analysis (CFA)

We used data from the second split-half to conduct a CFA using the SPSS AMOS v.26 software. A previous study suggested that the minimum sample size to conduct a confirmatory factor analysis ranges from 3 to 20 times the number of the scale’s variables [66]. Therefore, we assumed a minimum sample of 130 participants needed to have enough statistical power based on a ratio of 10 participants per one item of the scale, which was exceeded in this subsample. Our intention was to test the MDDI model extracted from the EFA. Parameter estimates were obtained using the maximum likelihood method and fit indices. For this purpose, the normed model chi-square (χ^2^/df), the Steiger-Lind root mean square error of approximation (RMSEA), the Tucker-Lewis Index (TLI) and the comparative fit index (CFI). Values ≤ 3 for χ^2^/df, and ≤ 0.06 for RMSEA, and 0.90 for CFI and TLI indicate acceptable fit of the model to the data [67].

#### Measurement invariance by eating attitudes and weight status

To examine measurement invariance of the Ar-MDDI scores according to the eating attitudes, we conducted multi-group CFA [68] using the total sample. Measurement invariance was assessed at the configural, metric, and scalar levels [69]. Configural invariance implies that the latent MDD variable(s) and the pattern of loadings of the latent variable(s) on indicators are similar across eating attitudes and weight status (i.e., the unconstrained latent model should fit the data well in both groups). Metric invariance implies that the magnitude of the loadings is similar across eating attitudes and weight status; this is tested by comparing two nested models consisting of a baseline model and an invariance model. Lastly, scalar invariance implies that both the item loadings and item intercepts are similar across gender and is examined using the same nested-model comparison strategy as with metric invariance [68]. Following the recommendations of Cheung and Rensvold [70] and Chen [68], we accepted ΔCFI ≤ 0.010 and ΔRMSEA ≤ 0.015 or ΔSRMR ≤ 0.010 (0.030 for factorial invariance) as evidence of invariance. We aimed to test for eating attitudes and weight status differences on latent MDD scores using an independent-samples *t*-test only if scalar or partial scalar invariance were established.

#### Further analyses

Composite reliability in both subsamples was assessed using McDonald’s (1970) ω, with values greater than 0.70 reflecting adequate composite reliability [71]. McDonald’s ω was selected as a measure of composite reliability because of known problems with the use of Cronbach’s α (e.g., [72]). Finally, we examined the skewness and kurtosis values for the MDDI subscales scores, which were within defined range (skewness <|3|, kurtosis <|10|; [73]). Therefore, the sample was considered normally distributed. Consequently, Pearson correlation test was used to test the convergent and divergent validity of the Ar-MDDI scale with the other scores included in the study. Based on Cohen (1992) [74], values ≤ 0.10 were considered weak, ~ 0.30 were considered moderate, and ~ 0.50 were considered strong correlations. The latter analysis was done using SPSS software v.22.

## Results

### Exploratory factor analysis in the first split-half sample

Bartlett’s test of sphericity, χ^2^(78) = 813.7, *p* < 0.001, and KMO (0.786) indicated that the Ar-MDDI items had adequate common variance for factor analysis. The results of the EFA revealed three factors, which explained 57.68% of the common variance. The WRMR value was also adequate (= 0.079; 95% CI 0.065–0.087), indicating good fit of the model. McDonald’s ω was adequate for Factor 1 = Appearance intolerance (ω = 0.77), Factor 2 = Drive for size (ω = 0.71), and Factor 3 = Functional impairment (ω = 0.79).

### Confirmatory factor analyses

The fit indices of the three-factor model of the Ar-MDDI scale showed good results as follows: The Maximum Likelihood Chi-Square = 123.80 and Degrees of Freedom = 62, which gave a χ2/df = 2.00, TLI = 0.90, CFI = 0.92 and RMSEA = 0.073 [90% CI 0.054–0.091]. The standardized loading factors of the MDDI scale are summarized in Table 1. McDonald’s ω values were adequate for Factor 1 = Appearance intolerance (ω = 0.78), Factor 2 = Drive for size (ω = 0.69), and Factor 3 = Functional impairment (ω = 0.83). The standardized loading factors ranged from 0.50 to 0.85.

### Measurement invariance

254 (64.1%) of the participants had disordered eating attitudes (EAT scores ≥ 20). Indices (Table 2) suggested that configural and metric invariance were supported; but scalar invariance was not upheld. Thus, it is inappropriate to conduct further group comparisons. Moreover, 128 (32.4%) of the participants had abnormal BMI (BMI > 25). Indices suggested that configural, metric, and scalar invariance was supported according to the dichotomized BMI (Table 2). People with abnormal BMI scored higher in terms of appearance intolerance (11.82 ± 4.46 vs 10.44 ± 3.57; *t*(393) = 3.32; *p* = 0.001) and lower in terms of drive for size (7.23 ± 2.93 vs 7.87 ± 2.88; *t*(393) = 2.03; *p* = 0.044) than those with normal BMI. Finally, no significant difference was observed between the two groups in terms of functional impairment (8.56 ± 3.58 vs 8.19 ± 3.31; *t*(393) = 1.02; *p* = 0.310).

**Table 2:** Measurement Invariance according to eating attitudes and normal/abnormal body mass index in the total sample

Model	χ^2^	*df*	CFI	RMSEA	SRMR	Model comparison	Δχ^2^	ΔCFI	ΔRMSEA	ΔSRMR	Δ*df*	*p*
Eating attitudes												
Configural	294.43	124	0.890	0.059	0.096							
Metric	317.44	134	0.881	0.059	0.095	Configural versus metric	23.01	0.009	< 0.001	0.001	10	0.010
Scalar	389.29	145	0.842	0.065	0.095	Metric versus scalar	71.85	0.039	0.006	< 0.001	11	< 0.001
Body mass index												
Configural	266.18	124	0.905	0.054	0.066							
Metric	285.50	134	0.899	0.054	0.069	Configural versus metric	19.32	0.006	< 0.001	0.003	10	0.036
Scalar	309.79	145	0.890	0.054	0.070	Metric versus scalar	24.29	0.009	< 0.001	0.001	11	0.011

CFI = Comparative fit index; RMSEA = Steiger-Lind root mean square error of approximation; SRMR = Standardised root mean square residual

### Confirmatory analysis of the Big Three Perfectionism (BTP) scale using the total sample

Before conducting the analysis divergent validity, we ran a CFA on the BTP using the total sample to make sure that its three-factor structure is conserved in the Arabic version of the scale. The fit indices were adequate as follows: χ2/df = 370.48/101 = 3.67, SRMR = 0.049, TLI = 0.91, CFI = 0.93 and RMSEA = 0.082 [90% CI 0.073–0.091]. McDonald’s ω values were adequate for Factor 1 = rigid perfectionism (ω = 0.91), Factor 2 = self-critical perfectionism (ω = 0.88), and Factor 3 = narcissistic perfectionism (ω = 0.84).

### Divergent validity

Higher Ar-MDDI subscales scores were significantly associated with higher rigid, narcissistic perfectionism and self-critical perfectionism (except for drive for size). Older age was significantly associated with less drive for size. Finally, higher BMI was significantly associated with more functional impairment and lower drive for size (Table 3).

**Table 3:** Correlation matrix of continuous variables

	1	2	3	4	5	6	7	8	9
1. MDDI Functional impairment	1								
2. MDDI Drive for size	0.35***	1							
3. MDDI Appearance intolerance	0.31***	0.33***	1						
4. Eating attitude test	− 0.05	− 0.08	− 0.04	1					
5. Rigid perfectionism	0.28***	0.18***	0.22***	− 0.56***	1				
6. Self-critical perfectionism	0.38***	0.28***	0.17**	− 0.47***	0.72***	1			
7. Narcissistic perfectionism	0.17**	0.09	0.29***	− 0.35***	0.61***	0.58***	1		
8. Age	0.06	− 0.10*	− 0.01	0.03	− 0.11	0.02	0.04	1	
9. Body Mass Index	0.24***	− 0.19***	0.06	− 0.03	0.12*	0.14**	0.16**	0.28***	1

*p < 0.05; **p < 0.01; ***p < 0.001

MDDI = Muscle Dysmorphic Disorder Inventory

## Discussion

We conducted this study with the aim of validating the MDDI in Arabic in order to make it available for the Arabic-speaking populations around the world. Overall, our findings revealed that the validation of the Ar-MDDI yielded excellent properties in terms of factor structure, reliability, as well as convergent and divergent validity. Therefore, we preliminarily recommend its use for the assessment of MDD among Arabic-speaking men, at least in the Lebanese context.

CFA allowed the replication of the original three-factor structure of the MDDI. This three-factors structure has been originally highlighted by Hildebrandt et al. [47] and found in multiple validation papers tackling different populations (such as Brazilian women [75], American transgender men [76] [54], cisgender populations across distinct cultural settings [48–51, 77–81] and cisgender sexual minority populations [82])) was confirmed in our sample. In terms of reliability, the Cronbach’s alpha value of the Ar-MDDI obtained in the present study (0.81) corroborates the results of previous validation studies (e.g., the Brazilian [75], the Italian [83], and the German [78] versions), enabling us to suggest that the present Arabic versions of the MDDI seems to offer a reliable measure of MDD.

Beyond factorial validity and reliability, our findings revealed that perfectionism scores correlated positively with MDDI, which suggests divergent validity and align with previous evidence [42, 84–87]. The relationship between perfectionism and MDD is rather well-established [42, 84]. Perfectionism has even been suggested as a potential factor implicated in the development and maintenance of MDD, making affected individuals struggle to achieve an ideal body shape [88, 89].

Our data also provided preliminary evidence for the convergent validity of the Ar–MDDI scale, as it showed a significant correlation with eating attitudes. Our results are in agreement with a previous research [47]. Indeed, it has been shown that the traditional masculinity model is associated with more disordered eating [90]. Body ideals spread by sociocultural agents (parents, peers and media) incline to be internalized by the individuals, which can cause body image disturbances, MDD and the adoption of harmful behaviors such as restrictive eating, compulsive exercise, and others [91]. Furthermore, dysfunctional eating patterns significantly contribute to the onset and persistence of MDD [37, 88, 92]. MDD shares clinical features with ED symptoms, because of the presence of the compulsive exercise and rigid diet characterizing both disorders. In this line, a previous study revealed that orthorexia nervosa emerged as predictors of MDD symptoms in the bodybuilders [93].

### Limitations

Some limitations of the current work should be noted. First, since MDD could manifest among women (albeit with different features) [93–95], it is worthy to test the validity and reliability of the scale using women samples. Other validation studies are also required in specific groups, such as Arabic-speaking athletes and bodybuilders; given that this population is at a particularly heightened risk of MDD symptomatology and associated behaviors as compared to the non-bodybuilder population [42]. Second, the results of this study do not allow the generalizability of the results to other men from other age ranges. Third, the snowball technique and the unknown refusal rate predispose us to a selection bias. Fourth, other psychometric properties of the scales were not addressed in this paper (i.e. test–retest and convergent validity). Fifth, divergent validity was tested only using perfectionism measure.

## Conclusion

Our psychometric examination confirms the robustness of the Ar-MDDI, supporting the idea that the MDDI subscales evaluate three distinct elements of the psychopathology and manifestations of the MD. Functional impairment, which is required for the DSM-5 diagnosis of muscle dysmorphia, is not evaluated by the other commonly used assessment tools, supporting the main role of MDDI in screening for clinically relevant symptoms. Overall, our study provides a useful tool to assess muscle dysmorphia among Arabic-speaking men. Our Arabic version of the scale adds to the multitude of translated versions of this scale, allowing for cross-cultural comparisons of muscle dysmorphia among men. This would allow future clinicians to evaluate whether it is a public health concern in order to create appropriate strategies to reduce it.

## Supplementary Information

**Additional file 1. Appendix 1.** Arabic items of the Muscle Dysmorphic Disorder Inventory.

## Data Availability

All data generated or analyzed during this study are not publicly available due the restrictions from the ethics committee.

## Abbreviations

AR-MDDI: The Arabic version of the Muscle Dysmorphic Disorder Inventory
DSM-5: The Diagnostic and Statistical Manual of Mental Disorders, Fifth Edition
CFA: Confirmatory factor analysis
EFA: Exploratory factor analysis
MDDI: The Muscle Dysmorphic Disorder Inventory
MDD: Muscle dysmorphic disorder
