# Supplementary material for: Validation of the Arabic version of the Muscle Dysmorphic Disorder Inventory (Ar-MDDI) among Lebanese male university students
Source: J Eat Disord. 2023 Jan 26;11:11. doi: 10.1186/s40337-023-00737-8 (PMC9881329; doi:10.1186/s40337-023-00737-8)
Supplement: Supplementary file 1 — Additional file 1. Appendix 1. Arabic items of the Muscle Dysmorphic Disorder Inventory. [file 40337_2023_737_MOESM1_ESM.docx]

Appendix 1. Arabic items of the Muscle Dysmorphic Disorder Inventory.

|  | أبداً | القليل | بعض الأحيان | غالباً | دائماً |
| --- | --- | --- | --- | --- | --- |
| أعتقد أن جسدي نحيف / نحيف للغاية. |  |  |  |  |  |
| أرتدي ملابس فضفاضة حتى لا يرى الناس جسدي. |  |  |  |  |  |
| أكره جسدي. |  |  |  |  |  |
| أتمنى أن أكون أثقل. |  |  |  |  |  |
| أجد أن صدري صغير جدًا. |  |  |  |  |  |
| أعتقد أن ساقيّ نحيفتان للغاية. |  |  |  |  |  |
| أشعر أن لديّ الكثير من الدهون في الجسم. |  |  |  |  |  |
| أتمنى لو كانت ذراعي أقوى. |  |  |  |  |  |
| أشعر بالحرج من السماح للناس برؤيتي بدون قميص. |  |  |  |  |  |
| أشعر بالقلق عندما أتغيّب عن ممارسة الرياضة ليوم واحد أو أكثر. |  |  |  |  |  |
| ألغي الأنشطة الاجتماعية مع الأصدقاء (مثل مشاهدة كرة القدم، والدعوات لتناول العشاء ، والذهاب إلى السينما، وما إلى ذلك) بسبب جدول التمارين / التمارين. |  |  |  |  |  |
| أشعر بالاكتئاب عندما أتغيّب عن ممارسة الرياضة ليوم واحد أو أكثر. |  |  |  |  |  |
| أفتقد فرصًا لمقابلة أشخاص جدد بسبب جدول التمارين الخاص بي. |  |  |  |  |  |
